# Comparison of Genicular Nerve Block in Combination With Adductor Canal Block in Both Primary and Revision Total Knee Arthroplasty: A Retrospective Case Series

**DOI:** 10.7759/cureus.16712

**Published:** 2021-07-29

**Authors:** Promil Kukreja, Alana Venter, Lauren Mason, Alexander M Kofskey, Theresa Northern, Sameer Naranje, Elie Ghanem, Prentiss A Lawson, Hari Kalagara

**Affiliations:** 1 Anesthesiology and Perioperative Medicine, University of Alabama at Birmingham, Birmingham, USA; 2 Medicine, Edward Via College of Osteopathic Medicine, Birmingham, USA; 3 Medicine, University of Alabama at Birmingham School of Medicine, Birmingham, USA; 4 Department of Orthopaedic Surgery, University of Alabama at Birmingham, Birmingham, USA

**Keywords:** total knee arthroplasty, post-operative analgesia, adductor canal block, genicular nerve block, oral morphine equivalents

## Abstract

The management of pain in patients undergoing total knee arthroplasty (TKA) for chronic knee osteoarthritis (OA) has remained a challenge for the anesthesiologist regarding regional anesthesia as no single regional technique is adequate with regard to balancing effective analgesia with minimal muscle weakness. Severe postoperative pain following TKA has been shown to negatively impact patient outcomes and mortality. The genicular nerve block has recently been demonstrated to provide effective analgesia to the anterior and posterior knee capsule in recent studies. In this retrospective case series, we compare the efficacy of combined genicular nerve block (GNB) and adductor canal block (ACB) to only ACB in both primary and revision TKA patients. This combined novel approach for TKA patients can be utilized to improve patient pain scores and early ambulation, limiting the use of opioids and early discharge.

## Introduction

Chronic knee osteoarthritis (OA), an epidemic among aging populations, is a leading source of chronic pain in the United States and other developed countries [1]. The rise in prevalence of knee OA is a direct result of factors including longer life expectancy and increased body mass index (BMI), and it has been reported that at least 19% of American adults aged 45 years and older are affected [1,2]. Total knee arthroplasty (TKA) is a surgical procedure that provides relief for these patients suffering from severe pain and joint immobility related to knee OA when traditional conservative management has failed [3]. Weinstein et al. estimated that 52.2% of males and 50.6% of females diagnosed with symptomatic knee OA will undergo TKA in their lifetime [4]. TKA is the most common surgical procedure in the United States, and the rate continues to rise as it is projected that the number of surgeries will reach 3.48 million annually by the year 2030 [5]. Postoperative pain management following TKA remains a challenge for physicians as more than half of these patients experience extreme knee pain immediately post-surgery [5]. Knee pain associated with OA has been shown to be an independent risk factor for early mortality [6]. Therefore, reducing postoperative pain and early mobilization has become critical in reducing early mortality, preventing future chronic pain, and limiting the use of opioids [7].

Severe postoperative pain following TKA has been shown to negatively affect early mobilization, physical rehabilitation, time to discharge, and overall post-op recovery [3,8,9]. Balancing pain control with patient ambulation often proves to be a challenge due to both the motor blocking effects of many proximal nerve blocks and the often inadequate analgesia associated with more superficial periarticular injections [10]. Therefore, numerous modalities of analgesic treatment combining different methods of nerve blockade for effective postoperative analgesia and faster functional recovery have been discussed in the literature [5, 9-12]. However, the superior technique is yet to be determined.

Successful postoperative analgesia for TKA requires careful consideration of knee innervation, with an emphasis placed on branches of the femoral nerve innervating the anterior and medical aspects of the knee and branches of the sciatic nerve innervating the posterior aspects of the knee capsule [12]. In the context of nerve blockade, femoral nerve block (FNB) has traditionally been a critical component in the multimodal management of pain in TKA patients due to the high analgesic efficacy and the minimal risks associated. However, FNB is associated with decreased quadriceps motor strength leading to limited physical therapy (PT) participation, delayed ambulation, and discharge [13]. The adductor canal block (ACB) has been found to be an excellent alternative to the FNB, providing adequate analgesia to the anterior knee compartment while enabling an improved postoperative range of motion by sparing motor branches to the quadriceps muscles [9].

However, patients do not achieve adequate posterior knee analgesia as ACB pain relief is primarily limited to the anterior capsule of the knee. Infiltration between the popliteal artery and capsule of the posterior knee (IPACK) block is a relatively new regional anesthesia technique and has been shown to be a potentially superior method of motor-sparing pain control compared to prior techniques, providing analgesia to the posterior compartment of the knee without causing any motor weakness affecting ambulation [9,10]. The use of local infiltration analgesia (LIA) and surgeon-administered peri-articular injections (PAI) for TKA is another route of pain management that has gained popularity in recent years. These superficial methods may provide incomplete analgesia for patients postoperatively [13,14]. Most recently, the genicular nerve block (GNB) emerged as a novel intervention for alleviating both chronic knee and postoperative pain in TKA to provide adequate coverage of anterior knee capsule, medial, and superolateral aspects of the knee [10,13,15]. GNB and radiofrequency ablation of genicular nerves (RFGN) were originally introduced by Choi et al. as a therapeutic alternative for chronic knee OA and were quickly adopted for use in patients undergoing TKA due to the significant pain reduction and functional improvement observed in these patients [16]. GNBs target five main innervating branches of the knee, including the superomedial, inferomedial, superolateral, inferolateral genicular nerves, and the infrapatellar branch of the saphenous nerve. Several bony landmarks surrounding these nerves aid in proper needle placement under fluoroscopic guidance [15-17]. The GNB has a motor-sparing effect that is desirable for early ambulation postoperatively, better PT, and earlier discharge [10,13,18], making GNB the target of our comparative study to determine its role in the optimal management of TKA.

The comparison of combined GNB and ACB analgesia for TKA is currently lacking in the anesthesia literature. This retrospective case series investigates the efficacy of treating patients with a combined GNB and ACB approach compared to ACB alone in both primary and revision TKA patients. The authors hypothesize that the combination of GNB with ACB would provide superior analgesia, while also limiting motor weakness and improving postoperative pain score, ambulation, and oral morphine equivalents (OME) use in patients undergoing TKA for chronic knee OA.

## Materials and methods

This retrospective case series included patients undergoing primary or revision TKA at a tertiary academic medical center between July 1, 2019, and April 23, 2021. Eighty-two patients undergoing TKA provided written consent for the nerve block(s). Of these 82 patients, 52 (63.4%) underwent primary procedures, while 30 (36.6%) underwent revision procedures. Of the 52 primary TKA patients, 26 (50%) received only an ACB, while 26 (50%) received both an ACB plus genicular nerve blocks. Of the 30 revision TKA patients, 20 (66.7%) received only an ACB, while 10 (33.3%) received both an adductor canal and genicular nerve blocks. All nerve blocks were performed preoperatively with the patient in the supine position. The blocks were performed by a regional anesthesia fellow or senior resident under the direct supervision of an experienced regional anesthesia faculty. The primary or revision TKA was performed either under spinal or general anesthesia based on patient preference and medical indications. The visual analog scores of pain in the post-anesthesia care unit (PACU) and at 6, 12, 24, and 28 hours after surgery stop time were obtained. Cumulative OME were also calculated for 0-6 hours, 6-12 hours, and 12-24 hours postoperatively. In addition, ambulation distances were obtained for postoperative days 0, 1, and 2. SAS version 9.4 (SAS Institute Inc., Cary, NC) was used to conduct all statistical analyses. Data are expressed as means and standard errors (for continuous variables) or counts and percentages (for categorical variables). The Wilcoxon rank-sum test was used to compare OME, pain scores, and ambulation distance. Analyses were stratified by procedure type (primary vs revision). All tests of statistical significance were two-sided and used a significance level of 5%.

Ultrasound-guided technique for genicular block

**Figure 1:**
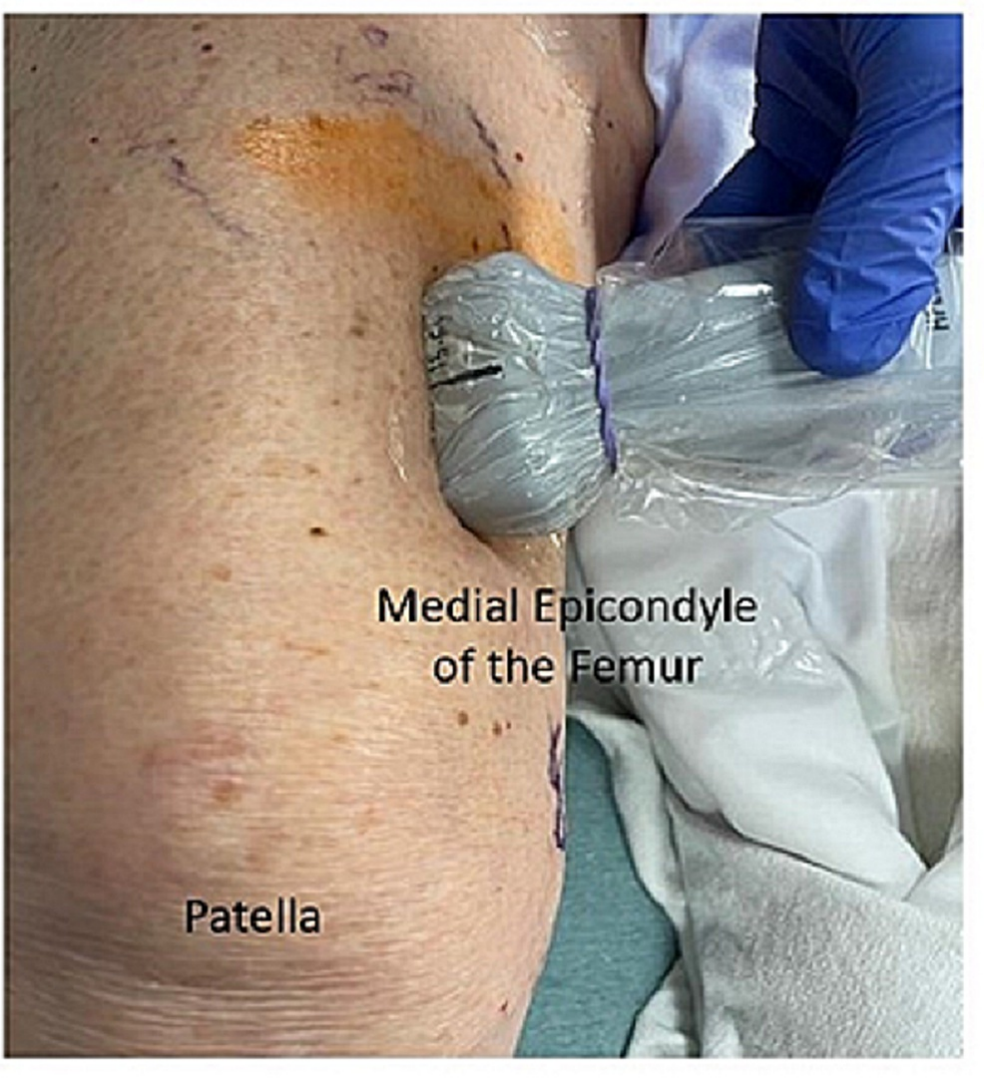
Probe positioning for superomedial genicular block The patient is in supine position with a linear probe placed along the longitudinal plane for femur.

**Figure 2:**
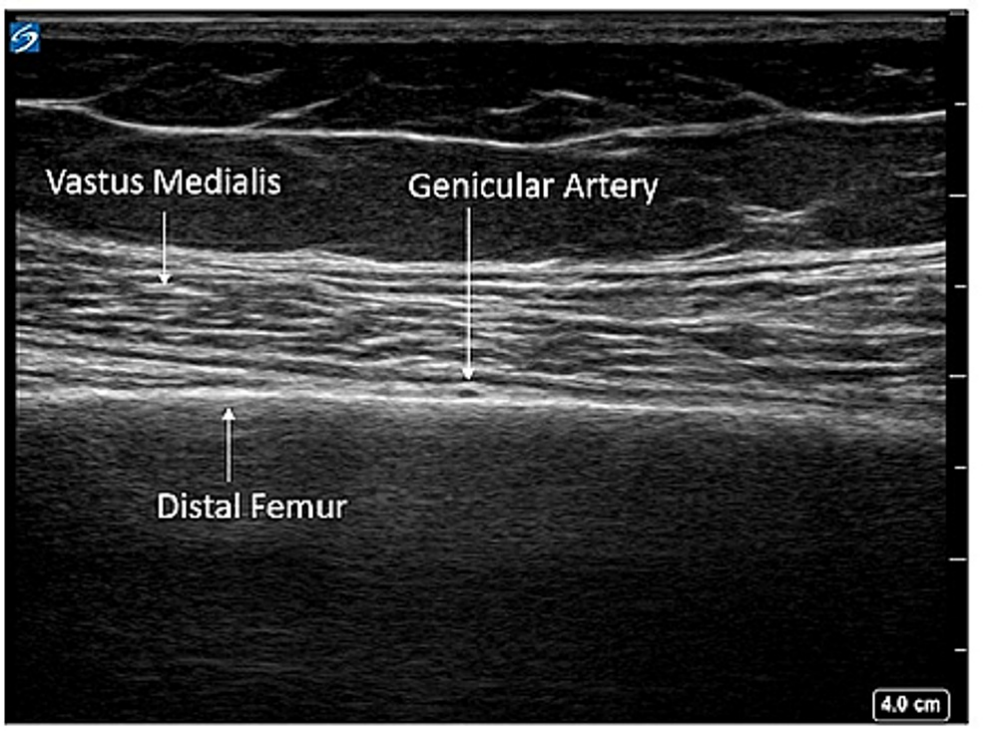
Superomedial genicular block anatomy To target the genicular nerve at this site, the genicular artery is used as a landmark along the distal femur.

**Figure 3:**
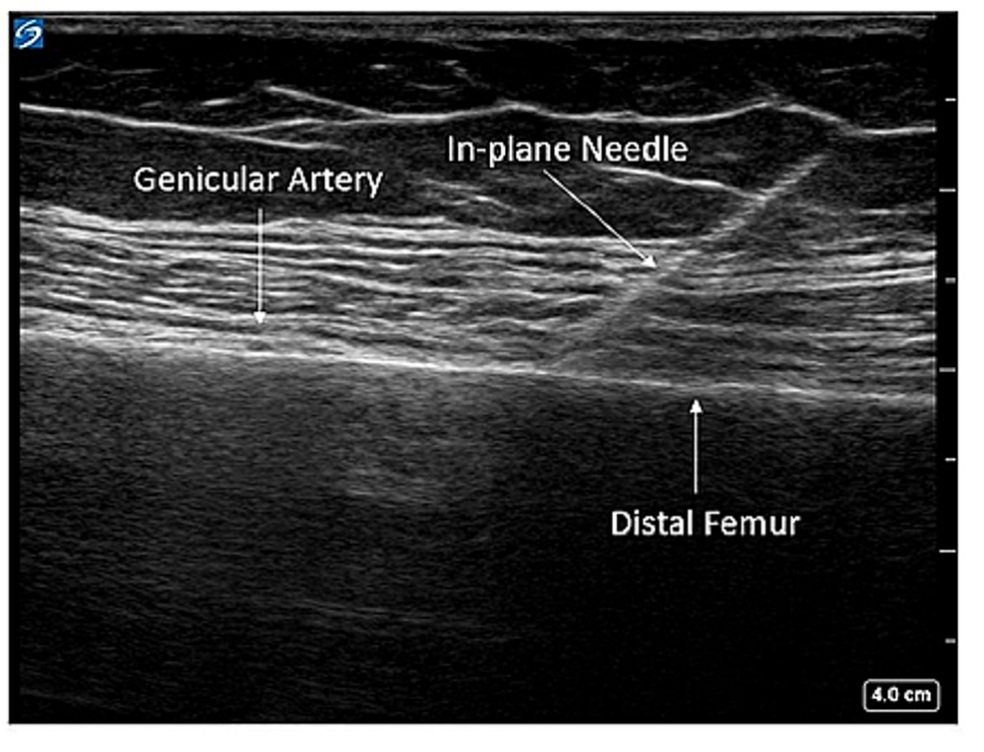
Superomedial genicular block with in-plane needle visualization Local anesthesia is injected along the periosteum near the genicular artery.

**Figure 4:**
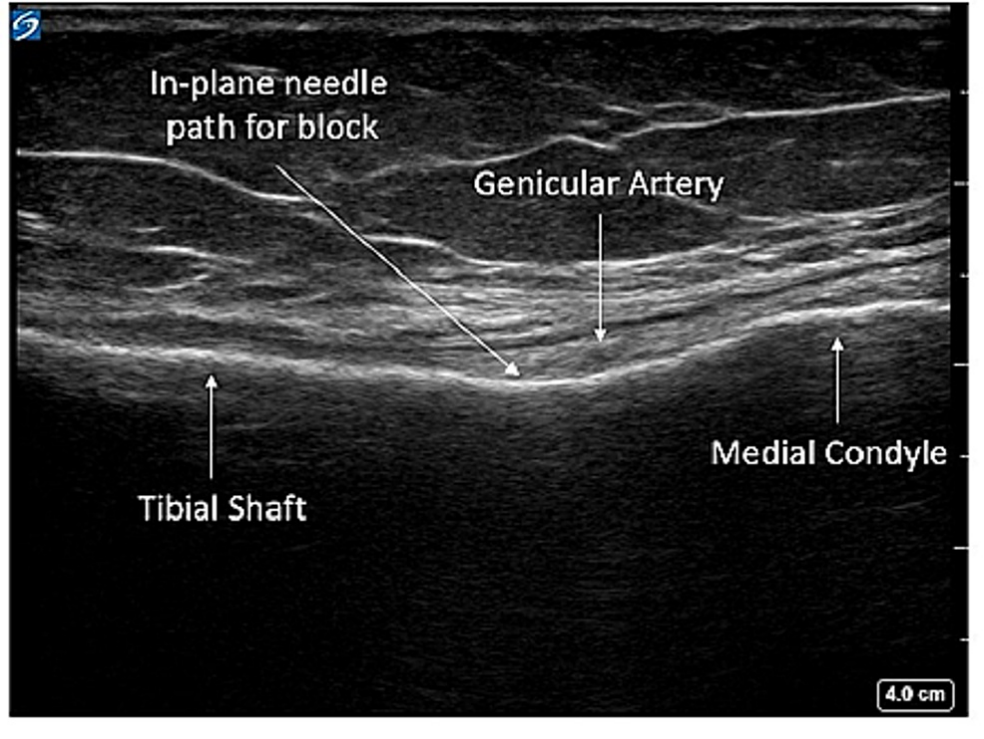
Inferomedial genicular block ultrasound anatomy Local anesthesia is ideally injected near the genicular artery (landmark) at the junction of medial condyle of tibia and tibial shaft.

The patient was positioned in the supine position, and a high-frequency linear transducer was used to target the superomedial, superolateral, and inferomedial genicular nerves. For the superomedial genicular nerve, a linear transducer was placed along the longitudinal plane of the femur to visualize vastus medialis, the distal femur, and the genicular artery (Figures 1, 2). Using an in-plane technique, a 10-cm echogenic 21-gauge needle was advanced in the cephalad to the caudad direction until the needle contacted femoral shaft, just cephalad to the genicular artery, and 5 ml of 0.5% ropivacaine was injected to spread along the femoral periosteum (Figure 3). This same technique, using 5 ml of 0.5% ropivacaine, was used in a mirror image on the lateral epicondyle to block the superolateral genicular nerve. To target the inferomedial genicular nerve, a linear transducer was placed in the sagittal plane on the anterior medial tibia. The genicular artery was identified, and a 10-cm echogenic 21-gauge needle was advanced in the caudad to the cephalad direction until bony contact was made. Five milliliters of 0.5% ropivacaine were deposited (Figure 4).

## Results

When considering only primary TKA patients, average pain scores for the genicular plus ACB group were lower across all recorded time points during the study (Table 1), with a statistically significant p-value (0.001) at the six hours postoperatively. In addition, for primary TKA patients, mean OME usage was also lower across all recorded time periods of the study with six- to 12-hour periods trending toward significance.

**Table 1:** OME, pain scores, and ambulation distance for primary TKA OME, Oral morphine equivalents; TKA, total knee arthroplasty; POD, postoperative day.

	Genicular Block (n = 26)	No Block (n = 26)	P
OME, mean (SE)			
0 to 6 h	18.57 (5.08)	27.61 (5.70)	0.139
6 to 12 h	9.13 (2.56)	13.32 (1.98)	0.053
12 to 24 h	14.18 (2.38)	19.62 (2.82)	0.206
Pain score, mean (SE)			
PACU	2.23 (0.69)	3.42 (0.79)	0.246
6 h	1.54 (0.54)	4.15 (0.58)	0.001
12 h	2.38 (0.70)	3.35 (0.67)	0.240
24 h	3.22 (0.59)	3.35 (0.58)	0.861
48 h	2.00 (0.68)	2.91 (0.67)	0.373
Ambulation distance, ft, mean (SE)			
POD 0	40.52 (12.93)	31.52 (9.31)	0.752
POD 1	116.22 (12.91)	94.92 (14.53)	0.098
POD 2	76.00 (15.76)	135.26 (19.23)	0.089

**Figure 5:**
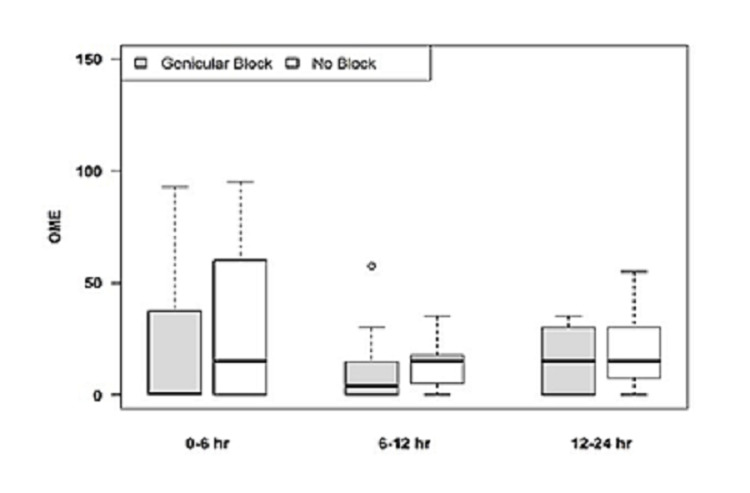
OME for ACB plus genicular blocks for primary TKA in postoperative period OME, Oral morphine equivalents; ACB, adductor canal block; TKA, total knee arthroplasty.

When comparing primary versus revision procedures for patients receiving genicular blocks, the average OME requirement was decreased in all time periods considered in the study for the primary procedure group, with a statistically significant reduction in OMEs at 0-6 hours postoperatively (p = 0.042) (Figure 5, Table 2).

**Table 2:** Comparison between primary and revision TKA patients with ACB plus genicular blocks ACB, adductor canal block; TKA, total knee arthroplasty; PACU, post-anesthesia care unit; POD, postoperative day.

	Primary (n = 26)	Revision (n = 10)	P
OME, mean (SE)			
0 to 6 h	18.57 (5.08)	35.90 (6.73)	0.042
6 to 12 h	9.13 (2.56)	15.88 (4.63)	0.112
12 to 24 h	14.18 (2.38)	22.25 (6.42)	0.196
Pain score, mean (SE)			
PACU	2.23 (0.69)	4.80 (1.14)	0.072
6 h	1.54 (0.54)	3.40 (1.01)	0.097
12 h	2.38 (0.70)	2.70 (1.00)	0.656
24 h	3.22 (0.59)	4.60 (1.07)	0.254
48 h	2.00 (0.68)	1.60 (0.81)	0.851
Ambulation distance, ft, mean (SE)			
POD 0	40.52 (12.93)	16.00 (6.54)	0.718
POD 1	116.22 (12.91)	150.30 (52.30)	0.829
POD 2	76.00 (15.76)	56.00 (20.64)	0.412

**Figure 6:**
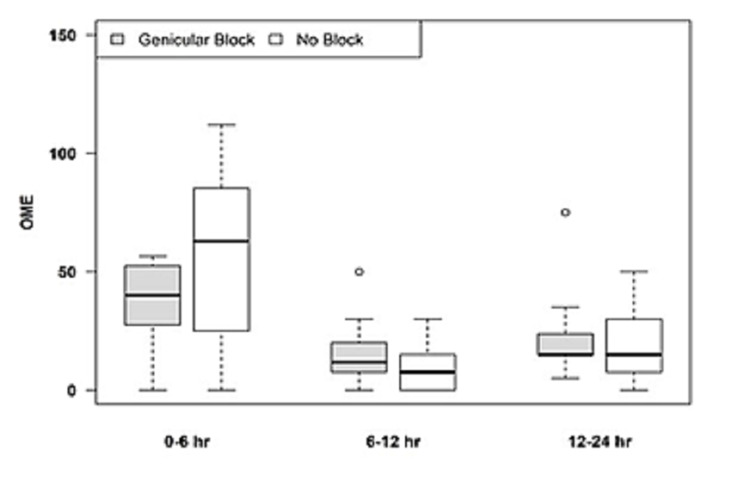
OME for ACB plus genicular blocks for revision TKA in postoperative period OME, Oral morphine equivalents; ACB, adductor canal block; TKA, total knee arthroplasty.

Revision TKA patients showed no statistically significant difference in OME requirements, pain scores, or ambulation distance with the addition of genicular nerve blocks as compared to ACB only (Figure 6, Table 3).

**Table 3:** OME, pain scores, and ambulation distance for revision TKA OME, Oral morphine equivalents; TKA, total knee arthroplasty; PACU, post-anesthesia care unit; POD, postoperative day.

	Genicular Block (n = 10)	No Block (n = 20)	P
OME, mean (SE)			
0 to 6 h	35.90 (6.73)	54.70 (7.89)	0.134
6 to 12 h	15.88 (4.63)	8.50 (2.25)	0.120
12 to 24 h	22.25 (6.42)	42.88 (25.46)	0.706
Pain score, mean (SE)			
PACU	4.80 (1.14)	5.55 (0.86)	0.560
6 h	3.40 (1.01)	4.45 (0.79)	0.457
12 h	2.70 (1.00)	2.80 (0.76)	0.981
24 h	4.60 (1.07)	2.70 (0.66)	0.135
48 h	1.60 (0.81)	2.58 (0.69)	0.497
Ambulation distance, ft, mean (SE)			
POD 0	16.00 (6.54)	20.25 (10.08)	0.710
POD 1	150.30 (52.30)	65.11 (16.53)	0.239
POD 2	56.00 (20.64)	68.46 (20.21)	0.921

**Figure 7:**
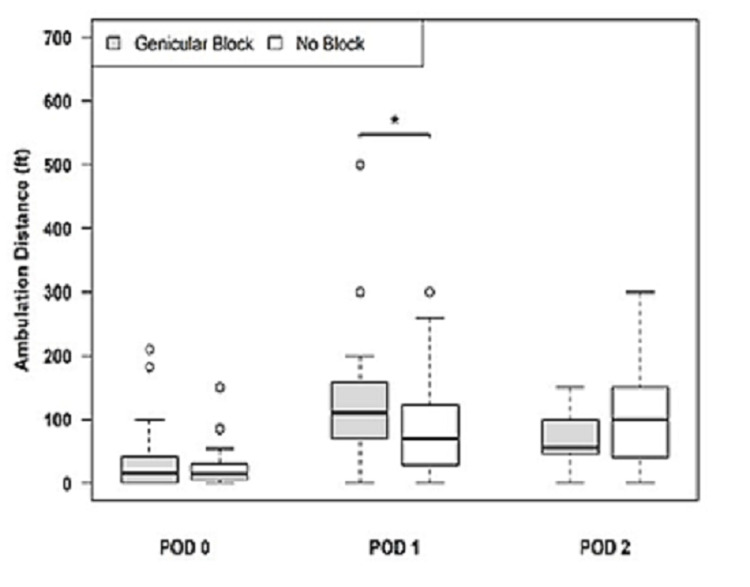
Ambulation distance for all procedures (primary and revision) with ACB and genicular blocks ACB, Adductor canal block.

When considering both primary and revision TKA, ambulation distance on postoperative day 1 (POD1) was significantly greater for patients receiving genicular nerve blocks, 126.55 feet (SD 17.91 ft), as compared to those who did not receive genicular nerve blocks, 82.55 feet (SD 11.03 ft) (p = 0.028). There were no statistically significant differences in ambulation distances for either POD0 or POD2 (Figure 7).

## Discussion

The clinical decision of a particular regional anesthesia technique should be based on the knowledge gained from the surgical anatomy and innervation of the knee. There is no single regional anesthesia technique, which can provide ideal analgesia without any motor weakness after TKA. All the peripheral nerve blocks and local infiltration techniques commonly used for postoperative analgesia have their own advantages and limitations.

The knee sensory innervation has been described as complex with significant anatomic variability [19]. Although the nerves supplying the knee originate from the lumbar (femoral and obturator) and sacral (sciatic) plexus, there is no consensus regarding the origin, distribution, and path of nerve branches supplying the knee capsule [20]. One of the cadaveric studies (Fonkoué et al.) provided distribution patterns and targeting of five genicular nerves [21]. This study also revealed that the nerve to vastus medialis, saphenous nerve, anterior branch of obturator nerve, and a branch from sciatic nerve supply significant innervation to the medial knee capsule and retinaculum. The sciatic nerve and the nerve to the vastus lateralis supply superolateral aspect of the knee joint, whereas the fibular nerve supplies its inferolateral quadrant. The tibial nerve and posterior branch of the obturator nerve supply the posterior aspect of the knee capsule. The GNBs can potentially block articular branches to the knee and are motor sparing.

In our retrospective study, in the combination group (ACB plus GNB), we performed superolateral, superomedial, and inferomedial genicular blocks. The rationale to abandon inferolateral genicular block was to avoid any unwanted motor weakness or potential foot drop by inadvertently blocking the branch of the common peroneal nerve. The mid-thigh approach of the ACB targets the saphenous nerve, medial cutaneous femoral nerve, nerve to vastus medialis, and possibly the articular branches of the obturator nerve. The ACB has become widely popular, given its motor-sparing analgesia, but it does not provide a complete block of the knee [21]. The combination of ACB with genicular blocks could potentially give substantial coverage of the knee.

In this study, for the primary TKA group, average pain scores for the genicular plus ACB group were lower across all recorded time points during the study (Table 1), with a statistically significant p-value (0.001) at six hours postoperatively. In addition, for primary TKA patients, mean OME usage was also lower across all recorded time periods of the study with the six- to 12-hour period trending toward significance. The early and short benefit of adding GNB can be explained by the low volume of local anesthetic (LA) used, location of injection, anatomic variation, block technique, inconsistent distribution of LA, or dissipation of LA during surgical incision and tissue plane disruption. The early and short benefit of GNB can also be appreciated by lower opioid consumption.

When comparing primary versus revision procedures for patients receiving genicular blocks with ACB, the average OME requirement was decreased in all time periods considered in the study for the primary procedure group, with a statistically significant reduction in OMEs at 0-6 hours postoperatively (p = 0.042) (Table 2). In this study, the combination of GNB with ACB resulted in better pain scores and lower opioid consumption during the early phase of recovery, which also resulted in better ambulation. When considering both primary and revision TKA, the ambulation distance on postoperative day 1 (POD1) was significantly greater for patients receiving genicular nerve blocks (Figure 7).

There are some limitations to this case series such as small sample size, retrospective design, and publication bias [22]. The complex innervation of the knee and variation in anatomical planes where nerves run could explain the inconsistency of block results [18]. The provider variability, site of injection, unfamiliarity with a new technique, and surgical technique could also affect the block results. For revision TKAs, there is a possibility of uneven spread of LA due to existing scar tissue and disrupted tissue planes.

The ideal regional technique for postoperative TKA management should provide effective analgesia with minimal muscle weakness to augment early functional recovery. At the same time, this case series explores a new combination approach for TKA patients, which can be utilized to provide effective analgesia and early ambulation. Large sample size studies are warranted to further understand this new technique and also to compare its efficacy with traditional blocks for knee analgesia. Also, cadaveric and magnetic resonance imaging studies are required for a better understanding of the anatomical spread of LA and nerves covered with various genicular blocks.

## Conclusions

The ACB plus genicular block provided adequate analgesia for patients undergoing primary TKA during the early postoperative period. This combination block for primary TKA reduced pain scores and had opioid-sparing effects postoperatively. The addition of genicular blocks helped improve early ambulation for both primary and revision TKA patients. The genicular block is an easy ultrasound-guided regional technique, which can be performed in the supine position for patient comfort. Further prospective randomized studies are warranted to determine the efficacy of genicular blocks for analgesia and quality of recovery after knee surgery. Also, the safety of genicular blocks alone or in combination with other blocks needs further investigation.
